# Blood transfusion practices affect CD4^+^ CD25^+^ FOXP3^+^ regulatory T cells/T helper-17 cells and the clinical outcome of geriatric patients with hip fracture

**DOI:** 10.18632/aging.203479

**Published:** 2021-09-01

**Authors:** Ling Wang, Wei Chen, Fu-Biao Kang, Ya-Hui Zhang, Li-Li Qi, Ying-Ze Zhang

**Affiliations:** 1 Department of Orthopedic Oncology, The Third Hospital of Hebei Medical University, Shijiazhuang, Hebei, PR China; 2 Department of Orthopedic Research Center, The Third Hospital of Hebei Medical University, Shijiazhuang, Hebei, PR China; 3 Department of Orthopedic Trauma, The Third Hospital of Hebei Medical University, Shijiazhuang, Hebei, PR China; 4 The Liver Disease Center of PLA, The 980th Hospital of PLA Joint Logistics Support Force, Shijiazhuang 050082, PR China; 5 Department of Pathogenic biology, Hebei Medical University, Shijiazhuang, Hebei, PR China

**Keywords:** regulatory T cells (Tregs), T helper-17 cells (Th-17), hip fracture, blood transfusion

## Abstract

Hip fracture (HF) is common among older individuals and associated with high mortality, poor vitality and functional impairment. HF patients suffer whole body immunological changes and that lead to severe consequences, including immobilization, physical impairment and a high risk of complications. The objective of this study was to decipher the pattern of dynamic immunological changes, especially in two major T cell subsets, CD4^+^ CD25^+^ FOXP3^+^ regulatory T (Treg) cells and T helper-17 (Th17) cells, and their balance, during the hospital stay and to observe whether blood transfusion could influence these cells and clinical patietns’ prognosis. In this study, ninety-eight consecutive HF patients were initially enrolled, and finally fifty-one patients qualified for the study, and correlation analysis of their clinical parameters was carried out to predict the meaning of their distribution in clinical practice. Our results showed that the frequency of Tregs gradually decreased, while the frequency of Th17 cells slowly increased in HF patients who received blood transfusion. The Treg frequency was inversely correlated with the level of hemoglobin (Hb), and Th17 cell frequency was positively related to fluctuations in Hb levels in HF patients after trauma. HF patients with a better prognosis and survival time showed decreased a Treg frequency and a decreased Treg/Th17 ratio. Transfusion helped reverse the imbalance in the frequencies of Tregs and Th17 cells and the Treg/Th17 ratio and especially contributed to a better outcome in HF patients with moderate-to-severe anemia. In conclusion, a higher frequency of peripheral blood Tregs and a higher Treg/Th17 ratio may be associated with unfavorable outcomes in HF patients, and blood transfusion may benefit moderate-to-severe HF patients rebalance their immune response.

## INTRODUCTION

Hip fracture (HF) is a major public health problem in the elderly population worldwide, that is associated with high morbidity and mortality. Gullberg et al. predicted that there will be more than 6.26 million HF patients in 2050, with almost half of them will exist in Asia [1–2]. The rates of HF occurring over the age of 50 years have increased to 58% in women and 49% in men in China according to Beijing statistics. Other cities in different countries have shared a similar increasing trend in recent years [3–4]. HFs have become almost the second most common cause of hospitalization of elderly patients, leading to high costs of medical care and an increased dependency on families [5–6]. Age, sex, body mass index (BMI), and bone mineral density (BMD) together with biochemical markers, such as hemoglobin (Hb), creatinine, potassium, and sodium, are associated with the occurrence and the outcome of HF. Therefore, better perioperative assessment of the condition of HF patients and prediction of their recovery status are urgently necessary in future treatment management plans for elderly patients.

Bone fracture repair is a multistage process that requires the participation of the skeletal and immune systems. Innate and adaptive immune responses play crucial roles in the entire bone-healing process [7–8]. For elderly HF patients, unsuccessful fracture healing and comorbidities often develop in the perioperative stage, which directly results in a negative final outcome in these patients despite careful medical and family care. Together with trauma, stress and age-related immune dysfunction could cause immunological suppression and a susceptibility to the development of post-traumatic infection [9–10]. However, the specific effects of HFs on the integral immune system are poorly documented. It is especially important to investigate the relationship between the immune response and the pathogenic mechanism of HFs. Mounting evidence indicates that Treg cells are involved in maintaining tolerance, regulating innate and adaptive immunity and promoting bone repair. Similarly, Th17 cells also induce osteoclast formation and regulate bone resorption via the activation of nuclear factor-κB ligand (RANKL) [10–11]. However, little is known about the dynamics of Treg and Th17 cells during the recovery phase in HF patients.

The present study investigated the correlation between the frequencies of Tregs and Th17 cells and the clinical traits of HF patients. For most HF patients, anemia is often accompanied by increased morbidity and mortality after repetitive surgery. Therefore, blood transfusions are frequently given to HF patients to save their lives. However, the effects of restrictive and liberal blood transfusion strategies on the outcomes of HF patients are controversial. We also performed expression analysis of the dynamics of Tregs and Th17 cells and the ratio of Treg/Th17 cells in HF patients who received blood transfusions. We analyzed the association between these immune cells and the clinical outcomes of these patients to predict the recovery status of HF patients.

## MATERIALS AND METHODS

### Study population and clinical criteria

All individuals successively admitted to the trauma emergency department from January 2019 to August 2019 were evaluated for eligibility and included when the primary admission was for HF and the individual was over 65 years in age. Patient data from the preoperative, intraoperative, and postoperative periods were accessible from the time the patient arrived at the hospital to postoperative day 30 in residential hospitals. The control population comprised 20 age-matched healthy individuals who were enrolled from the geriatric department. Trained surgical graduates collected all demographic data and comorbidities directly from the medical record and by interviewing clinicians involved in the unit. For patients with a Hb level of 8–10 g/dL, two units of red blood cells (RBCs) were normally given to help correct anemia, and four units of RBCs were given to patients with a Hb level of <8 g/dL. Blood was obtained from the blood transfusion department and given to patients within 30 minutes. The Ethical Review Board of the Third Hospital of Hebei Medical University in China approved this study. All individuals provided informed consent.

The following patient inclusion criteria were used: (I) patients aged 65 years or older; (II) patients volunteered to be involved in the study and signed the informed consent form; and (III) patients with HFs who were immediately admitted to the Department of Orthopedics at the Third Hospital of Hebei Medical University. The following exclusion criteria were used: (I) other compound injuries, such as severe cardiovascular disease; (II) multiple fractures or severe complications, such as vascular necrosis, inflammatory arthropathy, or infection around the knee, occurring before surgery; (III) bacterial and viral infections, such as acute bacterial inflammation or HBV/HCV/HIV infection; and (IV) autoimmune disorders and immunodeficiency diseases.

### Clinical assessment

The collected data included age, sex, BMI, medical comorbidity, fracture type, surgical delay, smoking status, alcohol consumption, and comorbidities (e.g., diabetes mellitus, hypertension, chronic heart disease, chronic pulmonary disease, chronic renal disease, liver cirrhosis, cerebrovascular disease, and tumors). Laboratory test results were also obtained from electronic medical records (EMRs) and included data on white blood cells (WBCs), RBCs, Hb, platelets (PLT), total protein (TP), albumin (ALB), and alkaline phosphatase (ALP). All patients received intramedullary nailing as the main treatment option. Two experienced radiologists assessed the X-ray results of the bone healing process and bone mass of the enrolled patients. The Harris hip score (HHS) system was introduced by William Harris in 1969 to evaluate the ability to conduct daily activities after hip surgery, and it is the primary pain assessment and functional activity index to evaluate the status of HF patients. HHSs were classified as follows to compare the different factors: < 70 poor; 70 to 79 fair; 80 to 89 good; and 90 to 100 excellent. The SF-36 score was also used to evaluate the recovery status of these patients.

### Blood sample collection

Blood samples were collected in heparinized tubes from the patients immediately after trauma induction (AT), before blood transfusion (when the patients received a transfusion) and after surgery. The samples were kept at 4°C for a maximum of 2 h, and peripheral blood mononuclear cells (PBMCs) were isolated for further flow cytometric analyses. We documented the values of laboratory indexes and immune cell analyses and divided them into two or more groups as needed, according to their reference range.

### Flow cytometry analysis

Purified PBMCs (100 μl) were collected into polystyrene fluorescence-activated cell sorting (FACS) tubes (BD Pharmingen™). To detect Tregs, 1 × 10^6^ PBMCs were surface stained with anti-human CD4-FITC (clone RPA-T4) and BV-605 mouse anti-human CD25 (clone 2A3) conjugated antibodies for 30 min at room temperature. The antibodies were washed twice, and the samples were treated with a fixation and permeabilization working solution. For intracellular staining, the samples were stained with anti-human Foxp3-PE (clone 259D/C7) or mouse IgG1 κ isotype (clone MOPC-21). To estimate Th17 cells, PBMCs were stimulated with a cell stimulation cocktail (eBioscience™, Cat# 00-4975-93) in complete culture medium (RPMI 1640 supplemented with 10% FBS) over 8 h at 37°C in 5% CO_2_. The cells were collected and surface stained with anti-human CD4-FITC or mouse IgG1 κ isotype control-FITC. After permeabilization, intracellular staining was performed with anti-human IL-17A-PE (BD, Cat#560438) or mouse IgG1 κ isotype control-BV421 (BD, Cat#562438) according to the manufacturer’s instructions. The plots showing CD25 vs. Foxp3 expression were obtained after gating CD3^+^ CD4^+^ T cells. The same gating criteria were used for the plots of CD4 vs. IL-17. Cells were measured using flow cytometry (BD celesta, USA). Data were analyzed using FlowJo software (Tree Star, Ashland, OR, USA).

### Statistical analysis

This study used SPSS version 20.0 (SPSS Inc., New York, USA) to perform all statistical analyses. For normal variables, the student’s *t*-test and one-way ANOVA were used to compare two groups and more than two groups, respectively. For non-normal variables, comparisons were performed between two groups and more than two groups using the Mann-Whitney *U*-test and Kruskal-Wallis test, respectively. The correlations between Treg and Th17 cells and clinical parameters were evaluated using Spearman’s or Pearson’s correlation analysis, respectively. The relationship between the frequency of Tregs and Th17 cells and the survival status was also investigated in this study. For this purpose, patients were categorized into two groups based on the median proportions of Tregs and Th17 cells (group 1 ≤ the median and group 2 > the median, respectively), and survival curves were plotted using Kaplan-Meier survival analysis. Differences for which *P* < 0.05 were considered statistically significant.

### Data availability

The datasets used and/or analyzed during the current study are available from the corresponding author on reasonable request.

## RESULTS

### Characteristics of the enrolled patients

**Figure 1:**
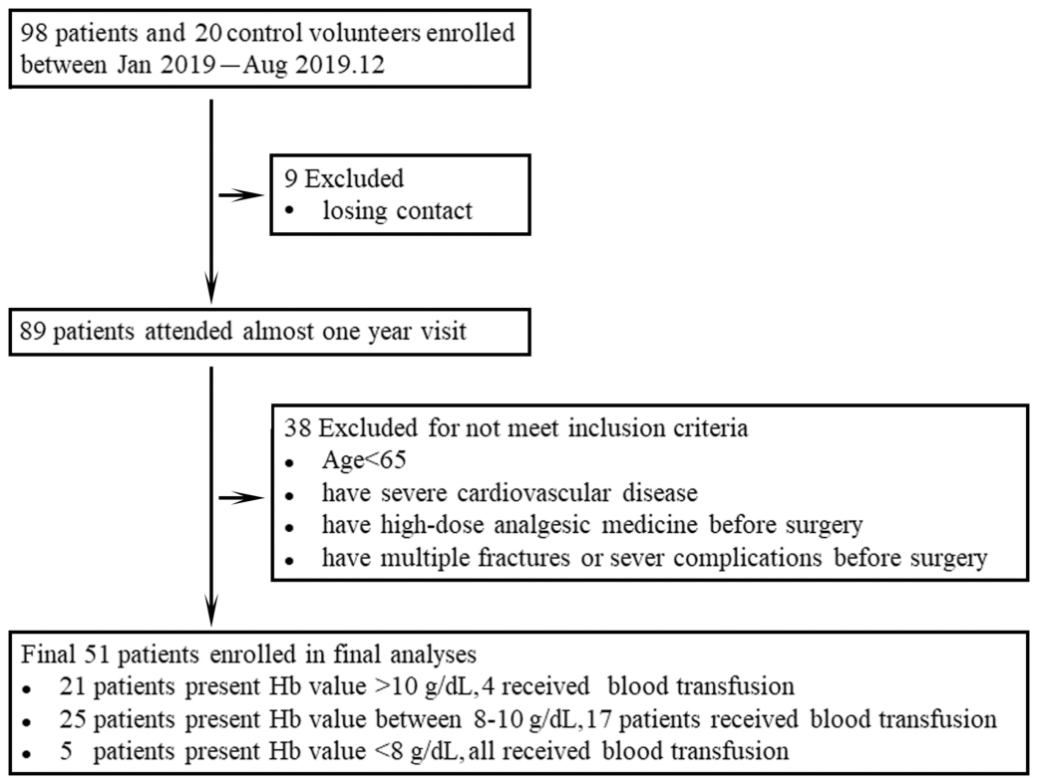
Data from all participants enrolled in the study are shown in the flow diagram.

To investigate whether dynamic changes in Treg and Th17 cells contribute to HF patient outcomes, 98 elderly patients were enrolled in our cohort beginning in 2019. Nine of these patients were excluded due to loss of contact, and 38 patients failed to meet the inclusion criteria. Overall, 51 patients and 20 healthy volunteers qualified for inclusion in the analyses (Figure 1). These patients consisted of 27 women (52.9%) and 24 men (47.1%) with a median age of 76 years (65–98 years). The baseline characteristics of the patients and schematic of the study are shown in Table 1.

**Table 1:** Patient demographics and comorbidities.

**Patient Demographics and Comorbidities**	
**Characteristic**	**Patient**
Age, mean (range)	76 (65–98) yrs
Sex, no (%)	
Female	52.9
Male	47.1
BMI, mean (range)	25.9 (18.6–35.7)
Mental status, no (%)	
Good	45 (88.2%)
Moderate	6 (11.8%)
Poor	0
Comorbidities, no (%)	
Hypertension	32 (62.7%)
Raised lipids	29 (56.8%)
Cerebrovascular disease	11 (21.6%)
Atrial fibrillation	3 (0.06%)
Deep vein thrombosis	4 (0.08%)
Hypothyroid	5 (0.09%)
Fracture type	
Intertrochanteric fracture	22 (43.1%)
Femoral neck fracture	29 (56.9%)
6-month mortality rate	9 (17.6%)

### Increased frequencies of Tregs in HF patients after injury

**Figure 2:**
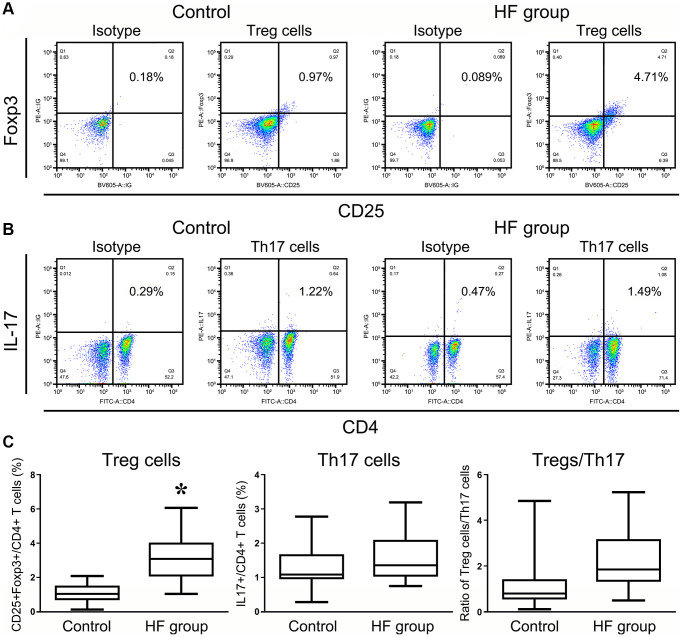
**Flow cytometry analysis of CD4^+^CD25^+^Foxp3^+^ Treg cells and CD4^+^IL17^+^ T cells in HF patients and healthy controls.** All of the values were gated on CD3^+^CD4^+^ T cells. The values shown in the each upper right quadrant represent the ratio of Treg cells (**A**) or Th17 cells (**B**) for the corresponding CD4^+^ cells. (**C**) The statistical histogram shows the changes in Tregs and Th17^+^ cells and the ratio of Treg/Th17 cells in HF patients and healthy controls.

Several studies have suggested that the roles of immune cells and their secreted cytokines are primarily involved in the regulation of the bone repair process. The immune functions of elderly individuals may be used as indicators for the future clinical treatment of HF patients according to the Senior European (SENIEUR) protocol [12]. Tregs and Th17 cells are associated with the response of the post-traumatic immune cascade, but the dynamics and mechanism are far from clear [13–14]. We examined the changing patterns of Tregs and Th17 cells in HF patients and analyzed the individual frequencies of these two cell populations and the ratio of Treg/Th17 cells using flow cytometry. As shown in Figure 2A–2B, the frequency of Tregs in PBMCs of HF patients was significantly higher than that in PBMCs of the healthy controls at 24 h posttrauma (*P* < 0.001), and some of these frequencies reached a 4- or 5-fold difference. The frequency of Th17 cells also showed an increasing trend compared with the healthy controls, but the trend was not significant (*P* < 0.005). For bone immune homeostasis, it is crucial to maintain an appropriate balance between Th17 and Tregs to avoid excessive inflammatory responses or immunosuppression [15]. Therefore, the ratio of Treg/Th17 cells was also detected and showed an ascending trend in HF patients after the first traumatic stress compared with the healthy control group, but the difference was not significant (*P* < 0.005, Figure 2C).

### The dynamic changing patterns of Treg and Th17 cell frequencies in HF patients before and after surgery

**Figure 3:**
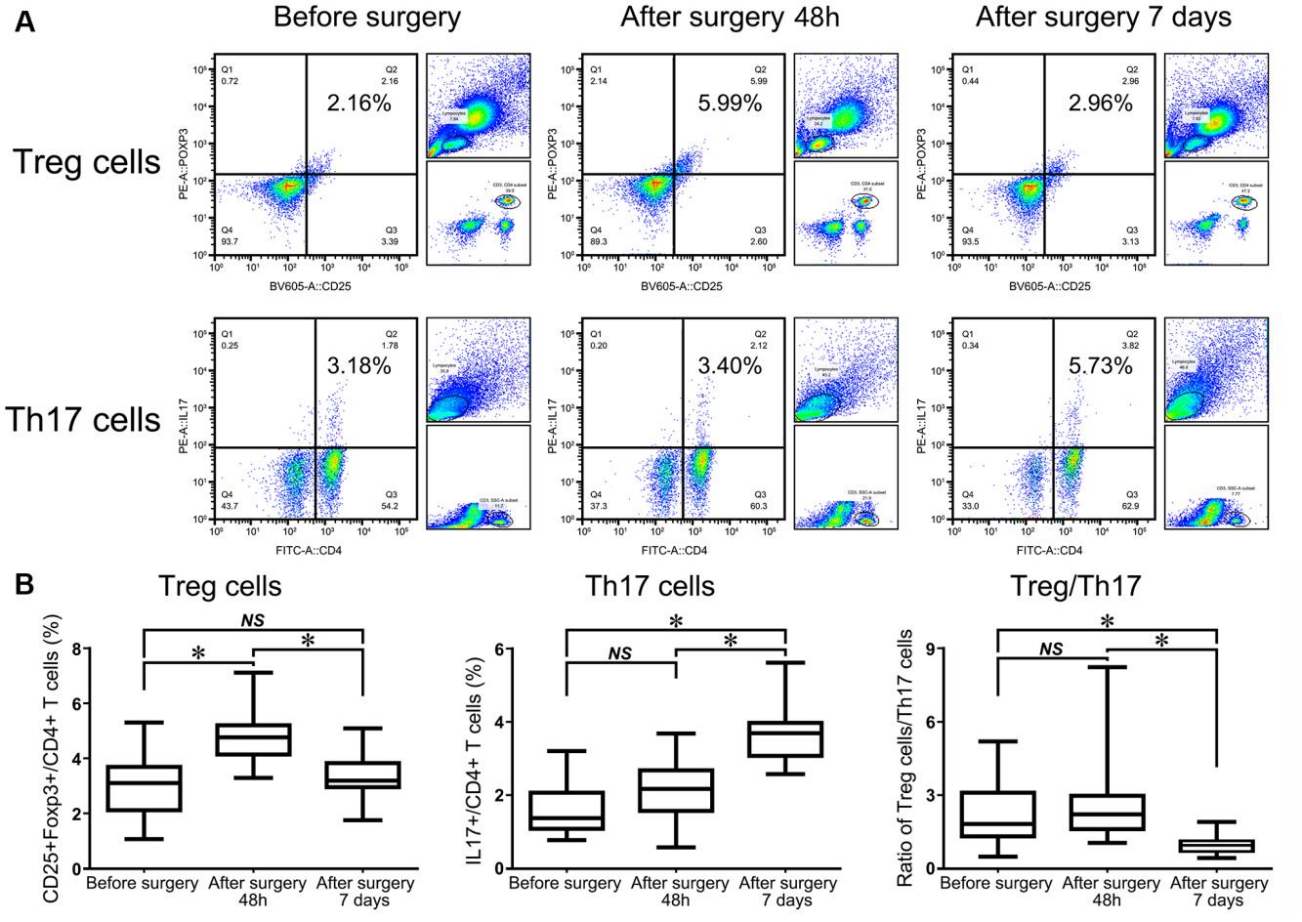
Flow cytometry analysis of CD4^+^CD25^+^Foxp3^+^ Tregs and CD4^+^IL17^+^ T cells in HF patients before surgery, 48 h after surgery, and 7 d after surgery (**A**). The statistical histogram shows the changes in Tregs, Th17^+^ cells and the ratio of Treg/Th17 cells in HF patients at the three different time points (**B**).

Bone healing is a complicated process that demands functional, immune-osteogenic cellular responses, which have become more elusive in elderly patients. Therefore, we evaluated the changing patterns of Tregs and Th17 cells in HF patients at different bone-healing stages before and after surgery. Our results revealed a significantly elevated number of Tregs in HF patients at 48 h after surgery despite undergoing a second stress caused by surgery, and this number gradually decreased within 7 d. However, the average frequencies of Tregs remained significantly increased compared with presurgery levels (Figure 3A).

Flow cytometry analysis of Th17 cells showed that the percentage of Th17 cells remained almost unchanged at 48 h after surgery compared with before surgery in the same patient (*P* = 0.201). However, the frequency of Th17 cells was significantly upregulated in patients in the observational period within 7 d compared with 48 h after and before surgery (*P* < 0.001, Figure 3A). The ratio of Treg/Th17 cells was slightly increased at 48 h after surgery compared with that before surgery in the same patient. However, the ratio began to dramatically decrease at day 7 compared with 48 h after surgery, which suggested that this lowering index had indicative functions in further evaluations of the patient prognosis (*P* < 0.001, Figure 3B).

### The relationship between the frequencies of Treg and Th17 cells and clinical and laboratory factors in HF patients

**Figure 4:**
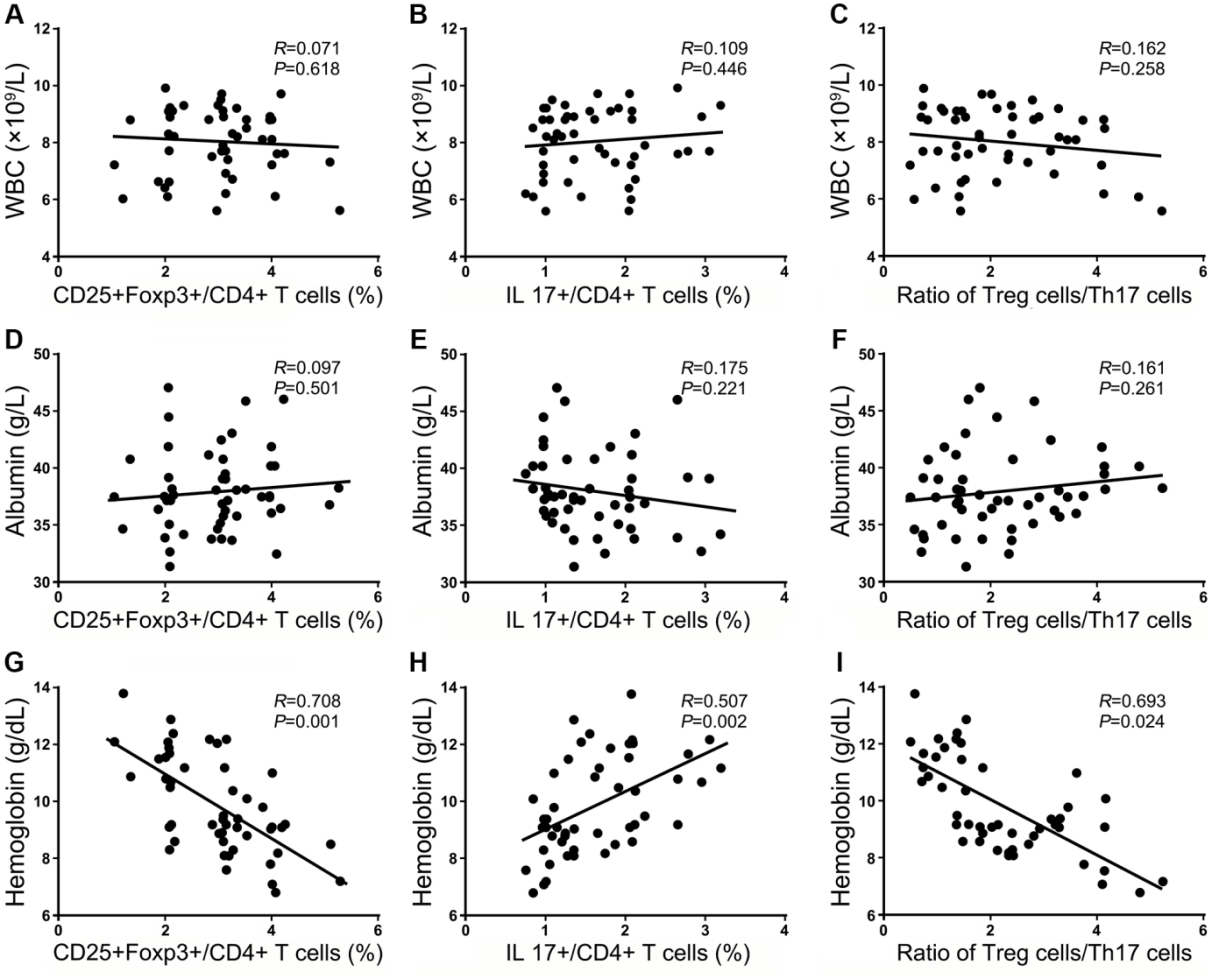
**Correlations between the frequencies of Tregs and Th17 cells and the ratio of Treg/Th17 cells with clinical laboratory parameters within 24 h after injury.** The correlation of Treg cells, Th17 cells, and the ratio of Treg/Th17 cells with the WBC count (**A**–**C**); the correlation of Treg cells, Th17^+^ cells, and the ratio of Treg/Th17 cells with the Hb level (**D**–**F**); the correlation of Treg cells, Th17 cells and the ratio of Treg/Th17 cells with the ALB level (**G**–**I**).

In numerous studies, potential factors have been searched for that contribute to the risk of mortality or other complications following HF surgery. A recent retrospective study found that the WBC count, PLT count, and Hb level were independent factors influencing mortality in HF patients [16]. Therefore, the frequencies of Treg and Th17 cells and the ratio of Treg/Th17 cells were evaluated for correlations with the clinical parameters in HF patients using Spearman’s rank correlation coefficient to compare the frequencies of these cell subsets and clinical parameters, including the WBC count and, ALB and Hb levels, in our cohort within 24 h after injury. No significant association was observed between most of the clinical laboratory parameters, including the WBC count, the frequencies of Treg and Th17 cells, or the ratio of Treg/Th17 cells (*P* < 0.05, Figure 4A–4C). Hb is released from RBCs into the peripheral blood circulation and may provide essential nutritional support to patients. Blood loss due to the double stress of trauma and surgery encountered in patients likely causes a devastating immune imbalance state, and abnormal disequilibrium of Treg/Th17 cells could aggravate this vicious cycle. Therefore, additional nutritional support that changes the Hb level may be an alternative treatment strategy to help restore the imbalanced immunological state. Among these indexes, ALB is a nutritional indicator that plays important roles in monitoring the nutrition level in HF patients. However, there was no correlation between ALB and these immunological factors, which suggests that the immune imbalance caused by anemia was not related to nutrition (*P* < 0.05, Figure 4D–4F). We also found that the frequency of Tregs and the ratio of Treg/Th17 cells were significantly inversely associated with the Hb level, and the frequency of Th17 cells positively correlated with the Hb level in HF patients, as shown in Figure 4G–4I.

### An elevated Treg frequency indicates impaired healing and a poor outcome for HF patients

**Figure 5:**
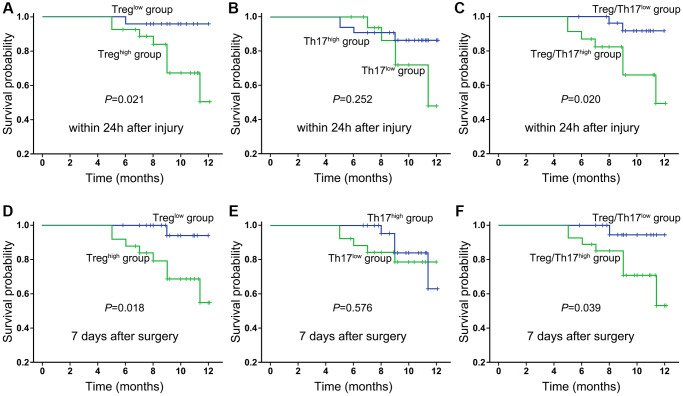
Kaplan-Meier survival curves of all enrolled HF patients in relation to Treg cells, Th17 cells and the ratio of Treg/Th17 cells at 24 h after injury (**A**–**C**) and day 7 after surgery (**D**–**F**). Differences between the strata were tested using log-rank statistics.

According to the latest statistics, nearly one-third of HF patients (29%) develop postoperative infections following surgery [17]. Postoperative complications represent some important factors associated with mortality. Therefore, we tested whether these immunological indexes contributed to the evaluation of prognosis and survival for HF patients. The results showed a trend toward an accumulation of Tregs in HF patients with a lower HHS, but this trend did not reach statistical significance. Similarly, the frequencies of Treg and Th17 cells or the ratio of Treg/Th17 cells were not associated with the SF-12 and SF-36 evaluation scores in HF patients after surgery (*P* < 0.05, respectively). The relationship between the frequency of Tregs and Th17 cells and the survival status was also investigated in this study. For this purpose, patients were categorized into two groups based on the median value for Tregs and Th17 cells (group 1 ≤ the median and group 2 > the median), and a survival curve was plotted using Kaplan-Meier survival analysis. Our results showed that the median frequencies of Tregs and the Treg/Th17 ratio (within 24 h after injury) were significantly higher in HF patients who had shorter survival times (Figure 5A–5C). On the day 7, the frequencies of Tregs and the Treg/Th17 ratio also exhibited potential indicative functions in predicting the prognosis of HF patients (Figure 5D–5F). A more in-depth analysis is presented in the following section on the use of transfusion as nutritional support in some of the enrolled patients.

### Transfusion alters the balance of Treg/Th17 cells in HF patients

**Figure 6:**
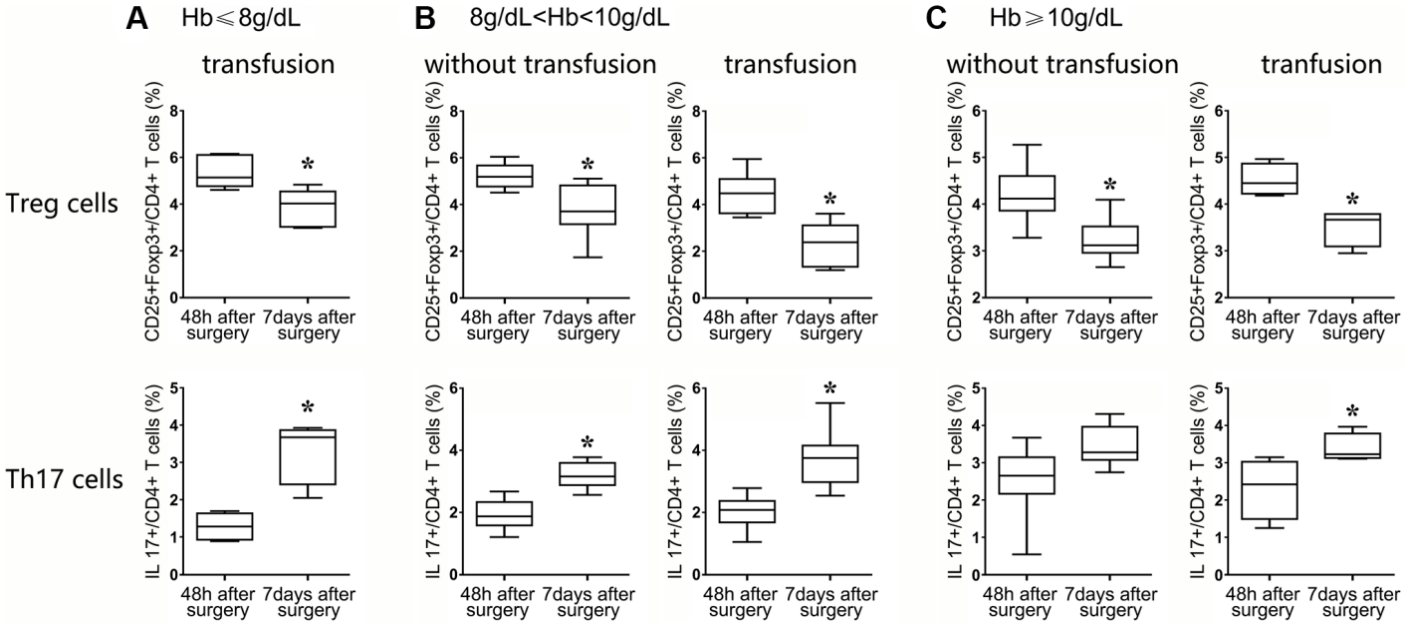
After surgery, the patients were classified into three groups: those with Hb ≤ 8 g/dL (**A**), 8 g/dL < Hb <10 g/dL (**B**), and Hb ≥10 g/dL (**C**). Flow cytometry statistical analysis histograms of CD4+CD25+Foxp3+Treg cells and CD4+ IL17+ T cells in HF patients stratified by transfusion or nontransfusion.

Because the Hb level was differently associated with the frequencies of Treg and Th17 cells, HF patients were divided into two groups (i.e., with or without transfusion before surgery) to evaluate the effects of transfusion. HF patients were categorized into 3 groups according to the level of Hb, Hb ≤8 g/dL, 8 g/dL < Hb <10 g/dL, and Hb ≥10 g/dL. Normally, all patients with Hb levels under 8 g/dL receive blood transfusions. For these patients, the frequencies of Tregs decreased by over 20%, and the frequencies of Th17 cells increased almost 30% (in the same patients) compared with the same indexes at 48 h and 7 d after surgery. Although these results were remarkably meaningful, it was difficult to demonstrate the benefit of blood transfusion in severely anemic HF patients due to the lack of a corresponding control group (Figure 6A).

Among patients with an Hb ranging from 8 to 10 g/dL, 17 received transfusion, and the remaining 8 patients did not receive transfusion. The Treg frequency was significantly decreased in both groups of patients at postoperative day 7 compared with the corresponding frequencies at postoperative at 48 h (*P* < 0.05, nontransfusion group; *P* < 0.01, transfusion group), but the degree to which the Treg frequency was reduced in the transfusion group was more obvious than that in the nontransfusion group (2-fold vs. 1.3-fold, *P* < 0.05). Similarly, the frequencies of Th17 cells in the transfusion group and the non-transfusion group were increased in a significant manner, but the degree of change at 7 d compared with 48 h in the two groups showed a significant difference, revealing a more significant upregulation in the transfusion group (2-fold vs. 1-fold, *P* < 0.01, Figure 6B). These data suggested that the Treg/Th17 ratio was more significantly decreased in the transfusion group than in the nontransfusion group (*P* < 0.01).

For patients with Hb levels over 10 g/dL, 4 patients received a blood transfusion due to a poor mental state, although the Treg frequencies of the transfusion and nontransfusion groups showed a decreasing trend between 48 h and 7 days after surgery (*P* < 0.05, nontransfusion group; *P* < 0.05, transfusion group), but the degree of change in the two groups of Tregs showed no significant difference (*P* = 0.543). Similarly, the upregulation of Th17 cell frequencies in the blood transfusion and nontransfusion groups between 48 h and 7 d after surgery also showed no significant difference (*P* = 0.319, Figure 6C).

### HF anemia patients with modest and severe symptoms benefit from blood transfusion before surgery

**Figure 7:**
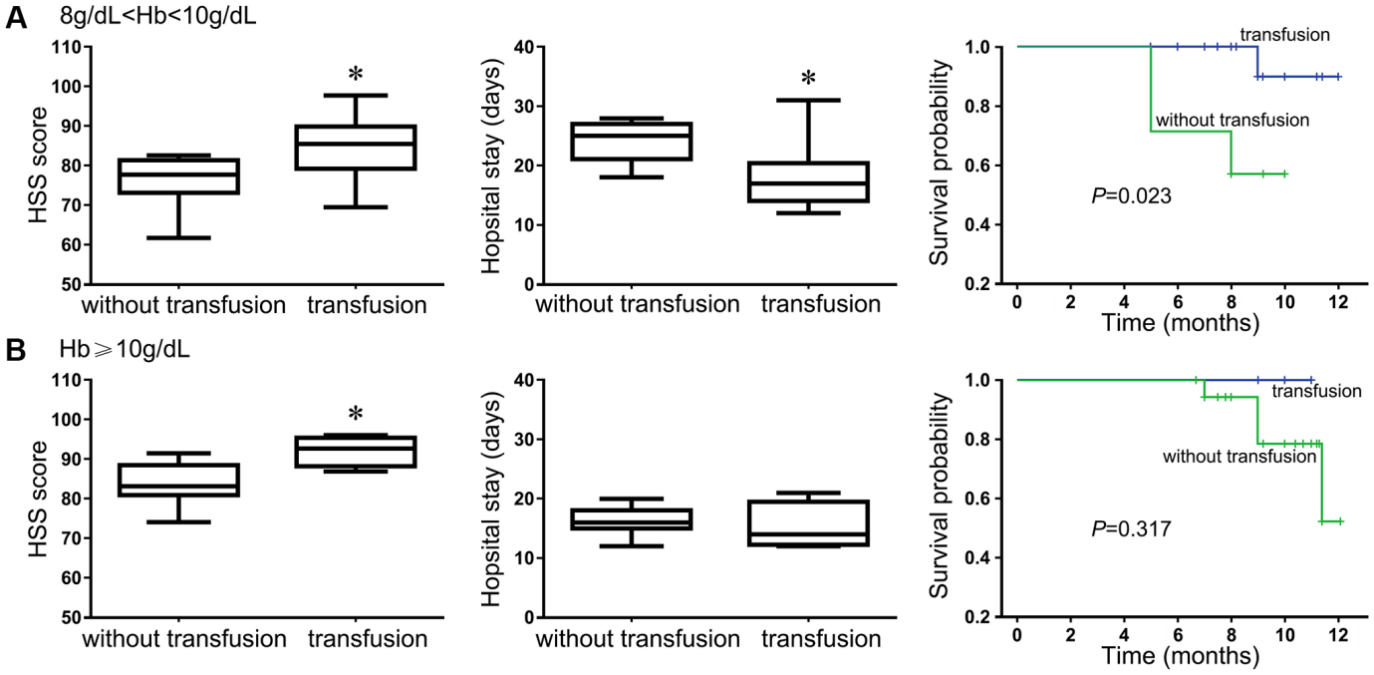
**The benefit of blood transfusion for HF patients was evaluated using the HSS score, hospital stay and 12-month survival outcome.** Patients with Hb level between 8 g/dL and 10 g/dL (**A**); Patients with Hb level equal or above 10 g/dL (**B**).

Our previous results showed that the Treg frequencies and the ratio of Treg/Th17 cells negatively correlated with survival and recovery after the evaluation of 12-month survival and recovery status in HF patients. Blood transfusion changed the state of immunological imbalance in trauma patients to some extent. Therefore, we further assessed whether blood transfusion could provide additional support for a shorter bed-rest period and in discharge mobility and self-care outcomes in patients after HF surgery. We noticed that the HHS score of the patients who received a blood transfusion improved significantly from 31.5 ± 6.7 (mean ± S.D.) preoperatively to 84.6 ± 9.8 in patients with Hb levels between 8 and 10 g/dL after definitive surgery. The average HSS score was significantly higher in those who received transfusion than in those without transfusion (*P* < 0.05, Figure 7A). Similarly, the average duration of hospital stay was also significantly shorter in patients who received transfusion than patients without transfusion (17.8 ± 3.7 vs. 23.9 ± 5.1, *P <* 0.005, Figure 7A). The survival status of patients with Hb levels between 8 and 10 g/dL was significantly longer when they received a blood transfusion than those without transfusion. However, for patients with Hb over 10 g/dL, the HSS score was also increased in the patients who received a transfusion, although only 4 of them did so voluntarily, however, the average duration of the hospital stay and the survival status were not significantly different from those of patients without blood transfusion (Figure 7B).

## DISCUSSION

According to recent statistics [18], population aging is expected to present a significant societal burden in China in the near future [19]. HFs constitute a major cause of excessive mortality and morbidity in the elderly, which intensifies the physiological and medical imbalances [4, 20]. The incidence of HFs increases by approximately 1–3% worldwide annually [21]. The immune system is altered in a significant manner due to deteriorating immune competence, which is associated with a higher susceptibility to infections, autoimmunity, and cancer in the elderly. After encountering HFs, acute inflammatory responses are initiated, which may result in additional organ damage and multiorgan failure with impaired T lymphocyte activation. However, the specific mechanisms of these dynamic alterations have not been completely elucidated.

CD4^+^ Tregs may increase with age, in the central lymphoid organs, peripheral blood and secondary lymphoid organs, and these cells exert an immunosuppressive function to inhibit monocyte differentiation into osteoclasts, which helps fracture repair [22, 23]. Interleukin (IL)-17 is a representative cytokine secreted by Th17 cells that stimulates osteoclast formation and activation via the upregulation of RANKL to further promote osteoclastic bone resorption [14, 24]. However, data on the roles of the frequencies of Treg and Th17 cells in HFs are scarce, especially in the elderly population. The frequencies of Tregs and Th17 cells were higher in HF patients after HF trauma than in the healthy, elderly controls in our study. There was also a statistically significant difference in the frequencies of Th17 cells between the controls and the patients. Therefore, the ratio of Treg/Th17 cells also showed remarkable changes in HF patients in the early post-traumatic stage. We found that the increased frequencies of Tregs began to decrease in a slow manner but remained higher than those of normal controls 7 d after surgery. Although Treg cells may help rebuild bone fractures, they may have devastating consequences for the host if they maintain intact repressive functions. Therefore, their frequencies should be appropriately restrained at a reasonable limit. Th17 cells, via the secretion of IL17, facilitate the maturation and maintenance of osteoblasts. Circulating Th17 cells in our HF patients were slightly increased after trauma and gradually climbed to almost 3–4 times the levels before surgery. Our findings are similar to those of Gupta et al. in trauma patients who developed sepsis. Because different publications have reported synergistic and antagonistic roles in the remodeling of bone fractures [14, 24], the ratio of Treg and Th17 cells may be a useful index to evaluate the changes if both cells are in the HF recovery stage. Notably, the change in Th17 cells did not present meaningful implications within 48 h after surgery. However, the Treg and/or Th17 frequencies showed important indications during each time point within the recovery stage of HF patients.

HF was recognized as the last event in traumatized elderly patients because of their increased susceptibility to infective complications related to their compromised and inappropriate immune reactions to trauma and surgery. However, there are few accurate indexes for evaluating the prognosis and survival status of HF patients. Therefore, we analyzed the relationship between the clinical laboratory indexes and the frequencies of Treg and Th17 cells and further examined the potential association with different recovery scores. The results showed that the frequency of Tregs and the ratio of Treg/Th17 cells increased with decreasing levels of Hb in HF patients. The high level of peripheral blood Tregs and ratio of Treg/Th17 cells may also be considered adverse prognostic factors for the prediction of disease outcome in these patients. Although this result is promising, this assumption should be addressed in a larger cohort of patients, especially patients for whom complete and definite follow-up information is available. Functional studies of how Treg and Th17 cells regulate the immunological response are necessary to elucidate the precise mechanisms underlying the different prognoses of HF patients.

HFs cause acute blood loss and lead to a reduction in Hb concentrations by approximately 7–25 g/L according to the type of fracture [25]. Several studies have reported a strong association between modest and severe anemia and reduced mobility and physiological reserve of patients upon admission after HFs. Therefore, some patients receive an allogeneic RBC transfusion based on their clinical conditions. However, there is no consensus on threshold values or when changes in postoperative Hb occur and stabilize. Blood transfusion has been an efficient way to increase the acceptance and prolonged survival of renal allografts since 1973 [26]. Some studies have shown that stored RBC transfusion induces Tregs and alters the balance between T cell subgroups [27–28]. However, the effects of blood transfusions on the adaptive and innate immunological systems remain controversial. The patients in our study were divided into three groups depending on their Hb level. We found that transfusion had a great influence on the frequencies of Treg and Th17 cells in patients with modest and severe symptoms of anemia, but there was a lack of controls for patients with Hb levels under 8 g/dL. As shown by the results, blood transfusion as an additional nutritional support ameliorated the trauma-induced immunological imbalance in critically injured patients. Appropriate transfusion may help patients recover in terms of shorter in-hospital stays, higher HHS scores and even a more energetic status. However, no significant benefit was observed in patients with Hb levels over 10 g/dL. Blood transfusion for clinical application remains controversial. Our study results suggest that only patients with modest or severe anemia benefit from blood transfusion, and the immunological cell types may be a predictable reference for considerations of blood transfusion.

In conclusion, we demonstrated dynamic changes in two main immune cell types, Treg and Th17 cells, in the trauma-healing process in HF patients. A shift in the normal balance may be detrimental to recovery outcomes and cause impaired/delayed healing. The imbalanced ratio of Treg/Th17 cells may contribute to suppression of the immune status. Appropriate nutritional support, such as blood transfusion, may be beneficial for moderate or severely ill patients when applied in a timely manner. However, more precise underlying immune-biological mechanisms should be elucidated and larger cohorts should be recruited in future clinical research.
